# Minding the Gap: Narrative Descriptions about Mental States Attenuate Parochial Empathy

**DOI:** 10.1371/journal.pone.0140838

**Published:** 2015-10-27

**Authors:** Emile G. Bruneau, Mina Cikara, Rebecca Saxe

**Affiliations:** 1 Department of Brain and Cognitive Sciences, MIT, Cambridge, MA, 02139, United States of America; 2 Department of Psychology, Harvard University, Cambridge, MA, 02139, United States of America; French National Centre for Scientific Research, FRANCE

## Abstract

In three experiments, we examine parochial empathy (feeling more empathy for in-group than out-group members) across novel group boundaries, and test whether we can mitigate parochial empathy with brief narrative descriptions. In the absence of individuating information, participants consistently report more empathy for members of their own assigned group than a competitive out-group. However, individualized descriptions of in-group and out-group targets significantly reduce parochial empathy by interfering with encoding of targets’ group membership. Finally, the descriptions that most effectively decrease parochial empathy are those that describe targets’ mental states. These results support the role of individuating information in ameliorating parochial empathy, suggest a mechanism for their action, and show that descriptions emphasizing targets’ mental states are particularly effective.

## Introduction

In the 20^th^ Century alone, over 230 million people were killed in the course of genocide, war, and other forms of group conflict [1], making intergroup conflict “one of the greatest problems facing the world today” [2]. While discord is driven by political factors such as competition over scarce resources and a history of violence, it can also be fanned by the flames of psychology. One of the psychological processes most commonly invoked in the context of intergroup conflict is (lack of) empathy [3–5].

While trait levels of empathic concern have previously been associated with interpersonal empathy, intergroup empathy has been shown to be dependent instead on the distribution of empathy, privileging in-group members over out-group members, which we have termed ‘parochial empathy’ (or intergroup empathy bias; [6]). Given that parochial empathy is associated with intergroup attitudes (e.g. positively with prejudice) and behaviors (e.g. negatively with outgroup altruism), understanding how to reduce the gap in empathic responding towards in-group and out-group targets may therefore be a critical step towards mitigating intergroup conflict.

Surprisingly few studies have examined directly the impact of interventions on intergroup empathy; however, previous work on stereotype reduction has shown that negative attributions (e.g. stereotypes) about out-group members can be reduced by providing individual-level rather than group-level information [7, 8]. Interestingly, stereotypes (e.g. a German engineer is efficient and socially withdrawn) can even be diluted by providing non-diagnostic information (e.g. that he likes his coffee with sugar) [9, 10]. In the current work, we extend this previous work on dilution in a number of ways. First, we predicted that descriptions about out-group individuals would dilute not only out-group perceptions, but also out-group affect, resulting in a reduction of parochial empathy. Second, we proposed a specific mechanism for this change: we predicted that narrative descriptions focusing on a target’s specific experiences and subjective perspective would reduce parochial empathy by decreasing the salience of that target’s group membership (i.e. social identity [11]), and focusing attention instead on the target as an individual human being. Third, we further predicted that not all information would work equally well—that the specific content of the narratives would influence their effectiveness at decreasing parochial empathy. Specifically, prior social cognition research has shown that a key component of being viewed as human is being endowed with a mind [12, 13]. In contrast, members of other groups are often denied a full human reasoning capacity, being likened to animals or automatons, a process known as dehumanization [14]. Dehumanization of an out-group in turn has been shown to predict failures in empathy towards that group [15]. Therefore, we hypothesized that narratives that endow out-group targets with uniquely human mental states would be particularly effective at mitigating parochial empathy.

Although short narratives about target individuals have been used previously to induce empathy, these studies almost exclusively examine empathy in the context of group stigma (e.g., the homeless, disabled, women with AIDS, convicted felons) rather than group conflict [16–18], and do not comment on what narrative qualities prove most effective. Surprisingly few studies have examined directly the impact of narratives on empathy between groups involved in zero-sum conflict. The current work therefore aimed to extend the previous research on perspective taking and empathy, as well.

### Current research

To create an intergroup empathy gap, we use an online paradigm to generate novel, competitive groups, and then measured people’s empathic responses to specific misfortunes and fortunes of individual members of those groups [6, 19]. A key advantage of using novel groups is that we can study the effect of group differences per se on empathy, distinct from potential effects of specific negative stereotypes about the out-group [20]; note, however, that parochial empathy findings replicate with real social groups (i.e., Americans versus Arabs; Bruneau et al., *in review*). Here, we operationalize empathy as self-reported congruent emotions (e.g. feeling bad when something bad happens to another person [21]), and parochial empathy as the difference in empathy reported towards in-group versus out-group members [22]. In Experiment 1, we characterize the impact of reading brief narrative descriptions about target individuals on parochial empathy expressed towards in-group and out-group members. In the subsequent experiments, we then test two hypotheses about the mechanism by which these narrative descriptions affect parochial empathy: by shifting participants’ attention away from the target’s group membership towards features of the individual (Experiment 2), and by drawing attention to the target’s mind (Experiment 3).

## Experiment 1: The Impact of Narrative Descriptions

In previous studies using the same paradigm used here [6], participants reported greater empathy for in-group relative to out-group fortunes and misfortunes encapsulated in single sentences. However, the sentences described relatively minor fortunes and misfortunes (e.g., missing a bus). In Experiment 1, we asked (1) whether the current paradigm could generate parochial empathy even for more significant fortunes/misfortunes (e.g. missing a flight to a best friend’s wedding), and critically, (2) whether providing brief narrative descriptions about the individual experiencing each event could decrease parochial empathy. We hypothesized that parochial empathy would be robust even in response to more consequential fortunes/misfortunes, and that narrative descriptions would specifically mitigate parochial empathy, rather than raise overall levels of empathy for all targets.

## Materials and Methods

### Participants

In Experiment 1, 720 American participants were recruited on Mechanical Turk. The study was designed as a 2 target group (ingroup versus outgroup targets) X 2 stimulus type (event-only versus event + description) X 2 event severity (mild versus extreme) X 2 identification order (identification questions before versus after the task) mixed design. Target group was repeated within participants; presence or absence of a narrative description, extremity of the events, and order of identification question administration were between participant manipulations. We subsequently decided to remain consistent with Study 2, so we present here the data only for the participants (*n* = 372) who received the intergroup bias questions prior to reading the scenarios (‘pre’). All subsequently reported main effects and interactions for pre participants were similar in post participants (*n* = 348) (S1 Table). Data from 50 pre participants who failed to pass the check question or catch scenario were excluded, resulting in 322 included participants (187 female, *M*_age_ = 31.5, *SD* = 11.0). Participants were randomly assigned to mild events-only, mild event + descriptions, extreme events-only, or extreme event + descriptions across two waves of data collection. Mechanical Turk participants come from various regions across the country. To ensure that we drew participants across conditions from the same subset of mTurk participants, we collected data for each condition at the same time of day (~ 12:00 pm EST on a weekday), when we presumed that workers on both the East and West Coasts would be represented. Participants were not allowed to participate in more than one version of the study; no participants attempted to do so.

Participants gave written consent to complete two tasks online: “in the first task you will read and rate other players’ experiences. In the second task you will complete a short problem solving challenge.” The study and consent procedures for Experiment 1 and all subsequent experiments were approved by the MIT Committee on the use of Humans as Experimental Subjects.

### Experimental design

#### Stimuli

Four stimulus sets, each describing in-group and out-group targets’ experiences (8 positive events and 8 negative events), were created for the study. Stimulus sets varied according to the severity of the events (mild or extreme), and narrative descriptions about each target that appeared before the events (events-only or events + description). In the event + description condition, the same events were presented following a short narrative description about the target (e.g. “Bryan is recently married and he is excitedly expecting the birth of his first child soon. Bryan has been working hard at a brand new job and is hoping to impress his boss so he can feel comfortable taking time off for paternity leave. Bryan worked furiously, trying to finish a big project before his child arrived. Just before his child's due date, Bryan's computer crashed. A full week of work was completely lost.”). Descriptive narratives were designed to contain the unitary dramatic structure of a Freytag Triangle (i.e. with a beginning, middle and end, and containing a rise and fall of tension [23]). The events were largely incidental to the preceding narratives. On a 100-point scale (0 = “mild”, 100 = “extreme”), an independent group of participants (*n* = 100) confirmed that the “mild” events were less extreme (*M* = 27.6, *SD* = 14.2) than the “extreme” events (*M* = 62.9, *SD* = 16.2; *t*(99) = 25.6, *p* < 0.001, *d* = 2.32) (stimuli in S1 File).

Participants were assigned to a team (either the ‘Eagles’ or the ‘Rattlers’), and saw all 16 stimuli from one of the four conditions (mild events-only, mild events + descriptions, extreme events-only, extreme events + descriptions). The scenario (e.g., “Andrew sat in gum on a park bench”) appeared below the logo written on a background that was either red (for Rattlers) or blue (for Eagles). The assignment of scenarios to in-group or out-group targets was counterbalanced across participants: each participant was presented with 4 in-group fortunes, 4 in-group misfortunes, 4 out-group fortunes and 4 out-group misfortunes. Scenarios were presented in a randomized order to each participant. A 17^th^ item (“Jack slipped, please push the sliders all the way to the right if you read this”) was included as an attention check.

The dependent variable (“empathy”) was the average congruent affect felt in response to target fortunes/misfortunes (i.e. the average of how bad participants felt about targets’ misfortunes, and how good participants felt about targets’ fortunes). Parochial empathy was the difference between in-group empathy and out-group empathy.

#### Procedure

Participants completed 5 items from the Big 5 Personality Inventory that were ostensibly used to assign them to a team. In fact, participants were randomly assigned to one of the two teams (the Eagles or the Rattlers), and told that the teams were competing in a problem solving challenge: whichever team successfully completed 100 tasks first would win extra cash ($1 per team member). Participants were told: “Scientific evidence suggests that the more people know about other players' personal experiences, the better people perform in these particular problem solving challenges. We're going to give you the opportunity to get to know the other players—RATTLERS and EAGLES team members—by letting you read some of their recent experiences… We would like you to tell us how each story makes you feel (using the slider bars below each story).” Immediately after assignment to a team, participants reported on 6 separate unmarked slider bars anchored at ‘strongly disagree’ and ‘strongly agree’ how much “I like”, “I value” and “I feel connected to” the in-group to which they were assigned (α = 0.89), and the out-group (α = 0.85). Responses for all 3 measures were converted to 100-point scales and averaged for each group to provide in-group and out-group identification measures; the difference between average in-group identification and average out-group identification provided an index of intergroup bias (IB). After this participants completed the 17 empathy items and reported demographic information.

## Results

Intergroup bias scores (range 32.5–37.7) were similar across all four conditions. A 2 group (in-group versus out-group) x 2 stimulus type (event-only versus event + description) x 2 event severity (mild versus extreme) mixed ANOVA revealed the expected main effect of group (*F*(1,318) = 446.1, *p* < 0.001, η^2^ = 0.58); there were no other significant main effects or interactions (*F*s < 1.0, *p*s > 0.30).

We had four predictions about parochial empathy in Experiment 1: we predicted a main effect of group (i.e. parochial empathy; Hypothesis 1) that would be qualified by an interaction with the presence or absence of a narrative description (parochial empathy would be reduced when narrative descriptions were present; Hypothesis 2). We also predicted a main effect of event severity (greater empathy for all targets in extreme relative to mild events; Hypothesis 3) that would not interact with target group membership, i.e. that parochial empathy would not be limited only to relatively inconsequential fortunes/misfortunes (Hypothesis 4). To test these predictions, we analyzed the empathy responses using a 2 group (in-group versus out-group) x 2 stimulus type (event-only versus narrative+event) x 2 event severity (mild, extreme) mixed-model ANOVA, with group as a within-subjects factor (for all main effects and interactions, see Table 1; see Table 2 for mean responses by condition).

**Table 1:** Main effects and interactions for Experiment 1. Results of the 2 group (in-group versus out-group) x 2 stimulus type (event-only versus event + description) x 2 event severity (mild versus extreme) ANOVA, with empathy as the dependent variable.

Effect	Hyp DF	Err DF	*F* Value	*p* Value	η^2^
Target Group (TG)	1	318	64.6	<0.001	0.17
Severity (SEV)	1	318	76.2	<0.001	0.19
Narrative (NAR)	1	318	4.8	0.030	0.02
TG X SEV	1	318	0.9	0.336	
TG X NAR	1	318	11.8	0.001	0.04
SEV x NAR	1	318	3.6	0.060	
TG X SEV x NAR	1	318	0.1	0.806	

**Table 2:** Average empathy by condition. Presented in each cell is mean empathy (±SD), for each group from all experiments, by condition.

	Experiment 1		Experiment 2		Experiment 3	
	Ingroup	Outgroup	Ingroup	Outgroup	Ingroup	Outgroup
Mild	*n = 91*		*n = 166*			
Event-only	67.8 (15.2)	55.4 (17.4)	65.8 (19.3)	54.0 (22.8)		
Mild	*n = 81*		*n = 168*			
Event+ nar	57.7 (18.7)	52.5 (19.0)	59.7 (16.3)	55.2 (18.0)		
Extreme	*n = 79*					
Event-only	77.5 (14.7)	67.5 (17.1)				
Extreme	*n = 71*					
Event+ nar	73.9 (14.0)	70.2 (16.5)				
Mild					*n = 94*	
Physical					61.9 (16.0)	54.1 (17.7)
Mild					*n = 93*	
Mental					60.7 (20.7)	56.4 (20.7)

**Fig 1:**
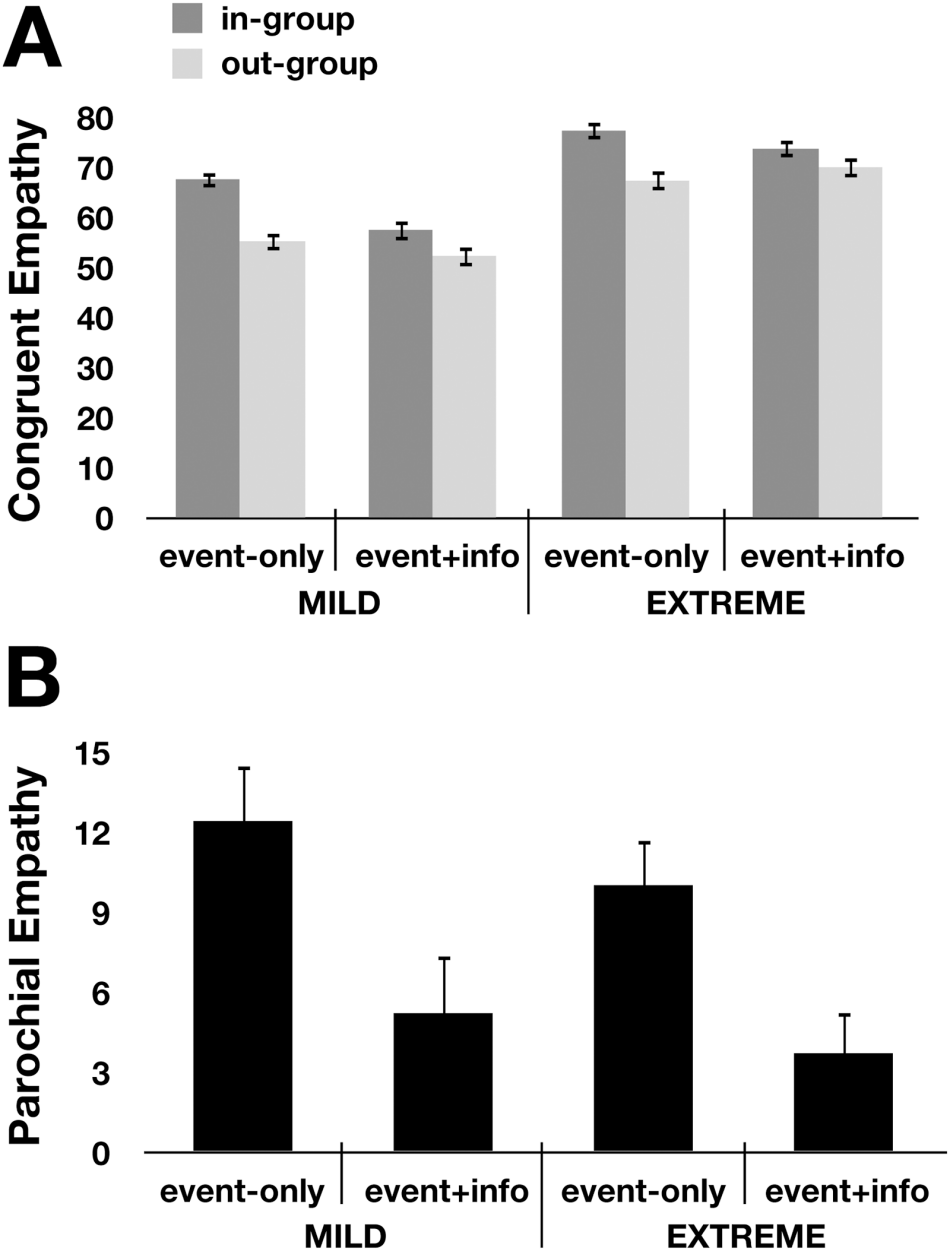
Narrative descriptions mitigate parochial empathy. (A) Empathic responses towards in-group targets (dark bars) and out-group targets (light bars) across each condition. Within-condition pairwise t-tests are reported. Error bars show standard error of the mean. (B) Parochial empathy (in-group empathy—out-group empathy) for each of the conditions presented in (a).

Confirming Hypothesis 1, and consistent with previous experiments [6], we found a significant main effect of group on empathy (*F*(1,318) = 64.6, *p* < 0.001, η^2^ = 0.169), with participants reporting higher empathy for in-group (*M* = 69.0, *SD* = 17.4) than out-group (*M* = 60.9, *SD* = 19.0) (Fig 1A). Confirming Hypothesis 2, the group x stimulus type interaction was also significant (*F*(1,318) = 11.8, *p* = 0.001, η^2^ = 0.036). Overall, mean parochial empathy in the event-only conditions (*M* = 11.3, *SD* = 17.6) was significantly reduced when narrative descriptions of the targets were provided (*M* = 4.5, *SD* = 17.1; Fig 1B).

The results also supported the last two hypotheses: confirming Hypothesis 3, there was a main effect of extremity (*F*(1,318) = 76.2, *p* < 0.001, η^2^ = 0.193), which did not interact with target group (Hypothesis 4; *F*(1,318) = 0.9, *p* = 0.034). As expected, extremity did not interact with the stimulus type (*F*(1,318) = 3.6, *p* = 0.060), and the group x stimulus type x severity interaction (*F*(1,318) = 0.1, *p* > 0.80) was not significant. All four hypotheses were supported also for counter-empathy (incongruent emotional responses: e.g., feeling good about negative events).

The only unexpected result was a small but significant main effect of stimulus type (*F*(1,318) = 4.8, *p* = 0.030, η^2^ = 0.015); surprisingly, adding narrative descriptions led to overall *lower* reports of empathy. We designed a second experiment to further investigate this unexpected result (S2 File).

## Discussion

First, Experiment 1 provided a replication of the results from previous work using this novel groups paradigm: empathic responses to in-group targets were significantly higher than responses to out-group targets [6]. These patterns replicated when reading about more extreme fortunes and misfortunes. Second, Experiment 1 confirmed our key hypothesis: adding a short narrative description about the target individual before each event reduced parochial empathy, for both mild and more extreme events. Third, we hypothesized that narratives reduce parochial empathy not by stimulating empathy, but by decreasing parochialism. Consistent with this hypothesis, the effect of narratives was not to increase empathy overall, but only to reduce parochialism (i.e. making empathy for out-group members more similar to empathy for in-group members).

Indeed, in an unexpected effect, narratives appeared to slightly reduce overall levels of empathy. To better understand this effect, we further investigated participants’ response to both event-only and event + description stimuli, outside of a group context (S2 File). When participants made judgments for individuals (in the absence of group membership information) the presence of the descriptions *decreased* both how good/bad the participants thought the targets themselves felt in response to fortunes/misfortunes and how good/bad they felt for the participants. Participants who read narrative descriptions may have weighed the specific events against the broader backdrop of the protagonist’s life, and thus predicted somewhat muted responses to the events when viewed in context (how much empathy you feel for someone losing $5 depends upon the context of the rest of their life—e.g. whether they are down on their luck or born with a silver spoon—which is revealed in the narrative descriptions).

Overall, results from Experiments 1 indicate that adding descriptive information about story targets interacted significantly with group membership, decreasing parochial empathy—but how? One possible mechanism through which descriptions could decrease parochial empathy is by ‘de-grouping’ targets. In addition to providing a broader context for specific events, narrative descriptions may focus empathic responses on the individual, rather than the group member, experiencing those events. We test this hypothesis directly in the next experiment.

## Experiment 2: Testing a Potential Mechanism of Narrative Impact on Parochial Empathy

In Experiment 1, we found that narrative descriptions decreased parochial empathy. The goal of Experiment 2 was to examine *how* descriptive information decreases parochial empathy. In the event-only conditions, participants were given little information about the targets other than group membership. In the absence of other information, decisions on how vigorously to engage empathic responses might be guided strongly by group membership. We hypothesized that the presence of descriptive information may shift attention away from each target’s group membership, and towards individuating information. To test this hypothesis, participants in Experiment 2 performed one of two unexpected two-alternative-forced-choice memory tests at the end of the experiment, recalling either each target’s group membership or the specific event each target experienced [24, 25]. We predicted that adding descriptive information would simultaneously degrade “group memory”, while leaving “event memory” intact (or improved).

## Method

### Participants

In Experiment 2, 400 American participants were recruited on Mechanical Turk, and each was placed in one of four conditions in our 2 task (‘group memory’, ‘event memory’) X 2 stimulus type (event-only, event + description) design; target group was a within-subject factor. Based on the same manipulation check and catch scenario as in the previous experiments, we excluded the data from 66 people. Of the remaining participants, 176 (97 female, *M*_age_ = 31.1, *SD* = 11.1) completed the ‘group memory’ task, and 158 (93 female, *M*_age_ = 30.2, *SD* = 10.8) completed the ‘event memory’ task. To ensure that we recruited from similar populations of participants across conditions, we collected data for each condition at the same time of day (~ 12:00 pm EST on a weekday); HITs were posted at least 24 hours apart from each other condition. Participants were not allowed to participate in more than 1 condition; no participants attempted to do so.

### Experimental design

The stimuli and experimental design were identical to Experiment 1, but were limited to only the mild events (events-only, events + description conditions). At the end of Experiment 2, participants were presented with a surprise two-alternative-forced-choice memory task, in which they had to determine either to which group each target belonged (“Group Memory”), or with which event each target was associated (“Event Memory”). Memory was used as an indicator of attention to that aspect of the stimuli [26, 27].

## Results

Intergroup bias (range 29.5–36.4) was similar across all four conditions: a 2 group (in-group, out-group) x 2 narrative information (event-only, event + description) x 2 task (group memory, event memory) mixed ANOVA, with group as a within-subject factor, revealed that beyond the expected main effect of group (*F*(1,330) = 384.8, *p* < 0.001, η^2^ = 0.54), there were no other significant main effects or interactions (*F*s < 2.1, *p*s > 0.15).

Experiment 2 had three main hypotheses. First, we predicted that descriptive information would decrease memory for targets’ group membership (Hypothesis 1). Second, we predicted that narrative descriptions would increase memory for targets’ individuating information (Hypothesis 2). Third, we predicted that the decrease in memory for group in the event + description versus event-only condition would mediate the effect of descriptions on parochial empathy (Hypothesis 3).

**Fig 2:**
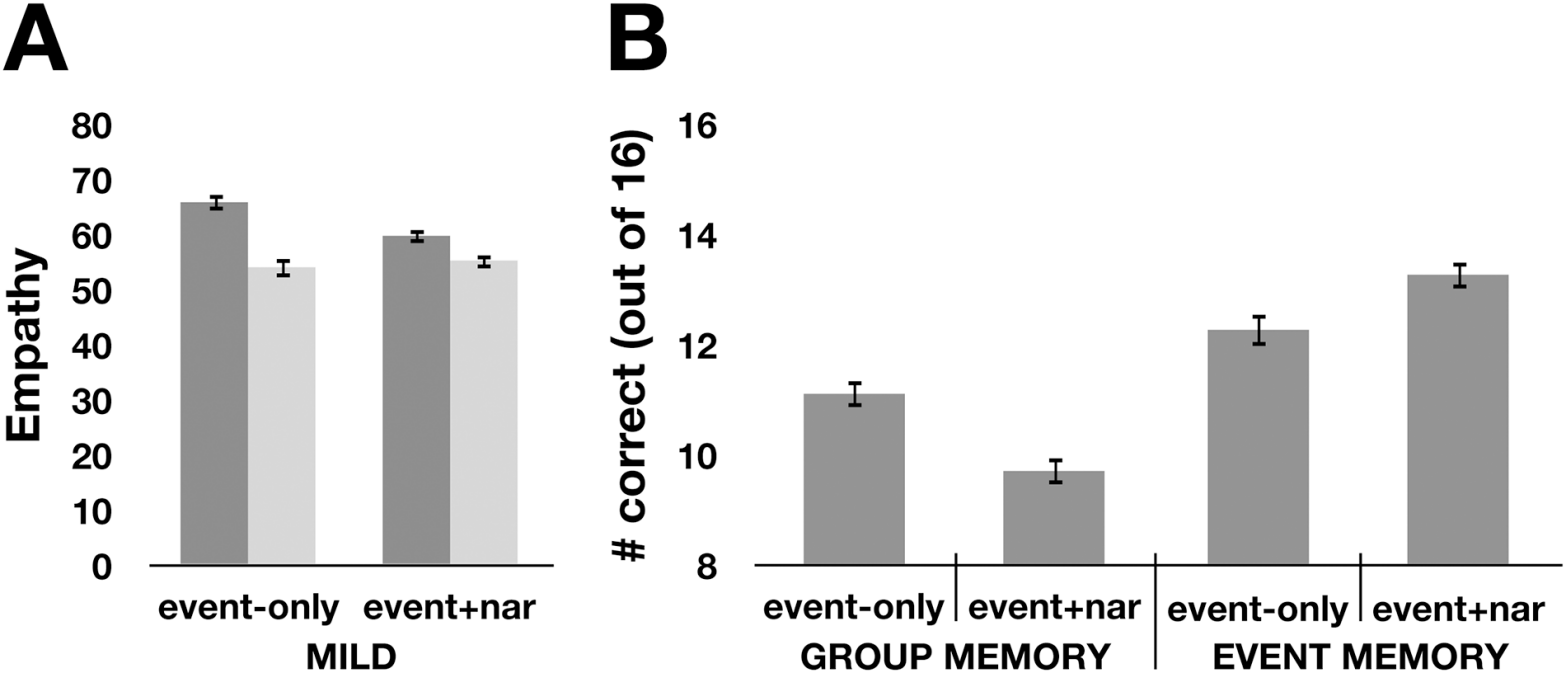
Narrative descriptions impair memory for group membership while improving memory for target-specific events. (A) Empathic responses to in-group targets (dark bars) and out-group targets (light bars) in response to mild fortunes/misfortunes presented by themselves (event-only) or after a narrative about the target (event+narrative). (B) At the end of the study, participants performed a 2-alternative forced-choice task to recall the group membership of each of the 16 targets (group memory), or the event that happened to each target (event memory). Error bars show standard error of the mean.

Prior to examining the targeted hypotheses, we first analyzed empathy responses using a 2 group (in-group, out-group) x 2 stimulus type (event-only, event + description) x 2 task (group memory, event memory) mixed ANOVA, with group as within-subject factor (see Table 2 for mean responses by condition). We found that there was no main effect of stimulus type (event-only versus event + description) or memory condition (group versus event) on empathy, but in line with our predictions, there was a main effect of group (greater empathy for in-group than out-group targets, *F*(1,330) = 70.9, *p* < 0.001, η^2^ = 0.18) that was qualified by a significant group x stimulus type interaction (*F*(1,330) = 14.4, *p* < 0.001, η^2^ = 0.04). Consistent with Experiment 1, the difference in empathy for in-group (*M* = 65.9, *SD* = 19.3) versus out-group (*M* = 54.0, *SD* = 22.8) in the event-only condition was significantly greater than the difference in empathy for the in-group (*M* = 59.7, *SD* = 16.3) versus out-group (*M* = 55.2, *SD* = 18.0) in the event + description condition (Fig 2A). No other interactions were significant.

We then turned to our planned comparisons in the memory data. To test Hypothesis 1, memory responses were analyzed using a 2 task (group memory, event memory) x 2 stimulus type (event-only, event + description) ANOVA. We found a main effect of task (*F*(1,330) = 88.8, *p* < 0.001, η^2^ = 0.04), which was driven by better performance on the event memory task (*M* = 12.8/16, *SD* = 2.4) versus the group memory task (*M* = 10.4/16, *SD* = 2.3). While there was no main effect for stimulus type, there was an interaction between memory task and description (*F*(1,330) = 22.5, *p* < 0.001, η^2^ = 0.06). To examine this interaction more closely, we turned to the simple effects within each memory condition: supporting Hypothesis 1, in the group memory task, participants who read events-only showed better memory for targets’ group membership (*M* = 11.1/16) than participants who read events + descriptions (*M* = 9.7/16; *t*(174) = 4.2, *p* < 0.001, *d* = 0.6; Fig 2B). Hypothesis 2 was also supported: in the event memory task, participants who read events-only had significantly *worse* memory for each target’s specific event (*M* = 12.3/16) than participants who read events + description (*M* = 13.3/16; *t*(156) = 2.5, *p* < 0.05, *d* = 0.4; Fig 2B).

**Fig 3:**
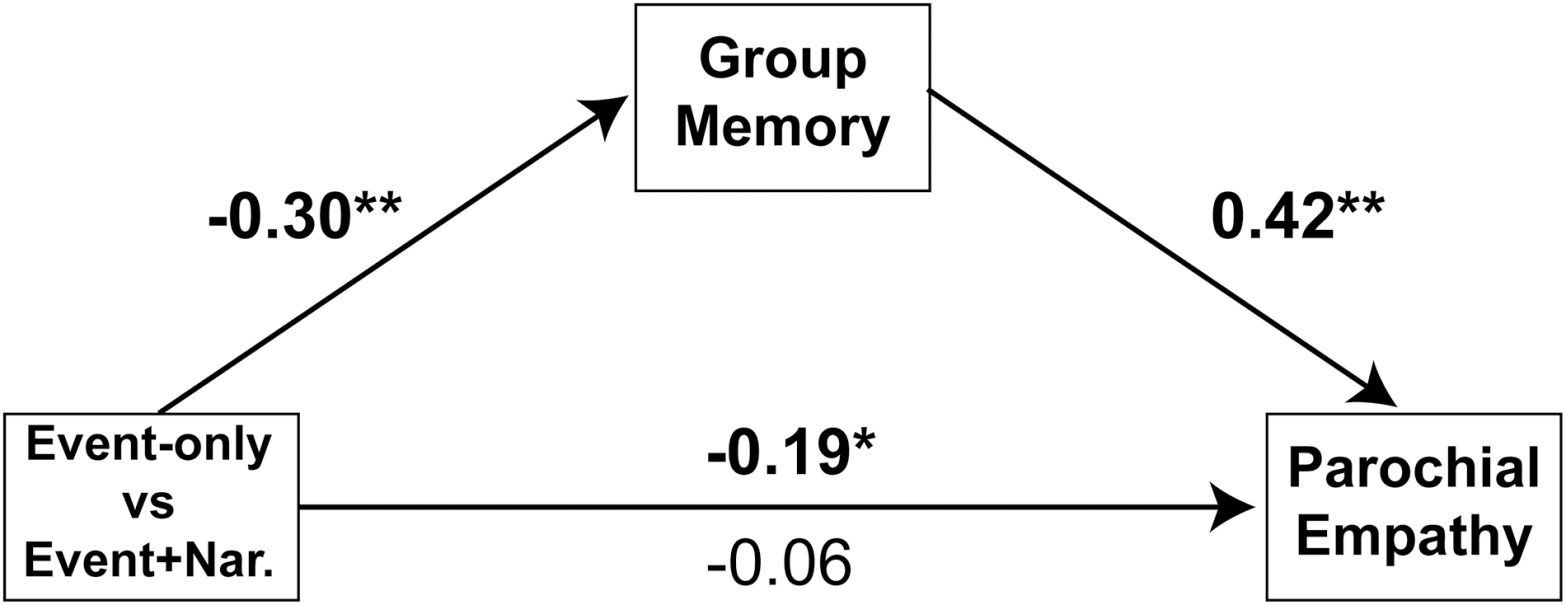
Memory for group membership mediates the relationship between condition (event-only versus event+narrative) and parochial empathy.

Finally, to determine if either Group Memory or Event Memory mediated the effect of the narrative descriptions on parochial empathy (Hypothesis 3), we conducted 2 separate bias-corrected bootstrap mediation analyses [28] using 5000 bootstrap samples, one for each task. This analysis revealed that memory for group membership mediated the relationship between description condition and parochial empathy (Sobel Test *z* = 3.3, *p* = 0.001; Fig 3). By contrast, memory for events did not mediate the relationship between story condition and parochial empathy (initial relationship: β = –0.22, *p* < 0.05; after controlling for event memory: β = –0.18, *p* < 0.05; Sobel Test *p* = 0.10).

## Discussion

We hypothesized that narrative descriptions decrease parochialism by reducing the salience of targets’ group membership, focusing attention instead on the targets’ experiences and perspectives. In support of this hypothesis, Experiment 2 showed that participants’ memory for group membership was worse after reading events + descriptions, relative to reading events only, and that this shift in attention mediated the effect of narrative descriptions on parochial empathy. Impaired memory for group membership in the event + description conditions occurred despite the fact that the descriptions take longer to read, and therefore required more exposure to group cues on the page (background color, verbal identification of group membership, group logo). At the same time, memory remained intact for other aspects of the individuals and events. In fact, memory for individuating information actually improved in the event + description condition, indicating that descriptions selectively shift attention from group membership to the specific individual.

Next, we tested two possible accounts of *how* descriptions reduce salience of the targets’ group membership.

## Experiment 3: Examining what types of descriptive information most effectively mitigates parochial empathy

Reading a short verbal description about an individual helps to mitigate the effects of competitive group membership on empathy by decreasing the perception of the target as a *group member* and increasing the perception of the target as a specific individual. How do these descriptions achieve this effect? That is, what aspects of an individual description make it an effective tool for ‘de-grouping’? Past research suggests that readers can be induced to lose themselves in narrative descriptions by manipulating the narrative structure. For example, narrative voice (first- versus third-person), the extent to which a written passage activates our ‘self-concept’, and when the identity of an out-group character’s identity is revealed, all affect the degree to which we take on the experience of the main character, and how we view that target character’s group [29]. In the present study we kept constant narrative voice and revelation of target identity, and instead varied descriptive content.

In Experiment 3, we explored the possibility that descriptions decrease parochial empathy by providing unique information about an individual’s characteristics and experiences—by individuating the targets. This additional information may dilute or distract from group identity as a salient characteristic [9]. If so, any information specific and unique to an individual would be equally effective for de-grouping. On the other hand, it is possible that narrative descriptions, which typically depict a sequence of events or experiences from a specific *perspective*, affect empathy by endowing the targets with a human mind. Since people have a tendency to deny ‘mind’ to out-group members [13, 30–32], and de-humanization predicts failures in out-group empathy [15], evoking the human mind of the protagonist may be particularly effective at decreasing parochial empathy.

To test this prediction, we generated two new stimulus sets: descriptions of the targets’ physical characteristics (“physical descriptions”) and descriptions of the targets’ personalities, thoughts and hopes (“mental descriptions”). We predicted that descriptions of targets’ minds would be more effective at humanizing them than descriptions of their physical appearances, and would therefore decrease parochial empathy the most.

## Method

### Participants

In Experiment 3, 200 American participants were recruited on Mechanical Turk, and each was randomly placed in one of two conditions (‘physical descriptions’ versus ‘mental descriptions’), with target group (ingroup versus outgroup) as a within-subjects factor. Based on the same manipulation check and catch scenario as in the previous experiments, data from 13 participants were excluded. Of the remaining participants, 94 (39 female, *M*_age_ = 29.3, SD = 9.6) completed the ‘Physical descriptions’ condition, and 93 (43 female, *M*_age_ = 30.2, SD = 10.8) completed the ‘Mental descriptions’ condition.

### Experimental design

In the ‘Physical descriptions’ condition, participants were told that each of the other participants had included a photograph of themselves, and an event that happened to them recently, and that these would each be described. These comprised the physical descriptions (e.g. “Melanie looks to be in her 20s. She has straight, blond hair that hangs down past her shoulders. She has a round face, a small nose and dimples in her cheeks. She is wearing shorts and a t-shirt with the sleeves rolled up. Recently, Melanie stepped in some dog poo.”). In the ‘Mental descriptions’ condition, participants were told that each of the other participants had sent in a brief description of themselves, along with an event that happened to them recently. These comprised the mental state narratives (e.g. “Melanie values creative thinking and loves ideas. She enjoys reading from a diverse set of thinkers, and likes to make connections between different ideas. She has always wanted to learn more about philosophy and is finally taking a class. Recently, Melanie stepped in some dog poo.”; stimuli in S1 File). The length of the physical descriptions (characters per narrative: *M* = 280.2, *SD* = 22.3) and the mental state descriptions (characters per narrative: *M* = 281.8, *SD* = 22.8) were matched (*t*(30) = 0.2, *p* > 0.8), and the time required to read and respond to the mental descriptions (*M* = 18.3 sec, 6.9 *SD*) and physical descriptions (*M* = 21.3 sec, *SD* = 10.4) was also similar (*t*(89) = 1.6, *p* = 0.10). The experimental design was identical to that used in Experiment 1.

Descriptions were also piloted in a separate sample online to determine the valence and engagement of each mental and physical description. Participants (*n* = 90) each saw a random half of the physical descriptions and a random half of the mental descriptions, and rated each on how positive/negative, and how engaging they were. Overall, mental descriptions were rated as more positively valenced (*t*(30) = 3.1, *p* = 0.004), and more engaging (*t*(30) = 2.8, *p* = 0.01) than physical descriptions. However, we were able to identify a subset of items (8 per condition) that were matched for valence (Mental *M* = 71.3, *SD* = 11.5; Physical *M* = 68.5, *SD* = 7.3; *t*(14) = 0.6, *p* = 0.60) and another subset of items that were matched for engagement (Mental *M* = 52.9, *SD* = 9.2; Physical *M* = 50.9, *SD* = 8.8; *t*(14) = 0.4, *p* = 0.70). After analyzing empathic responses to the full set of stimuli, we also analyzed empathic responses to in-group and out-group targets restricted only to the subsets of stimuli in the mental descriptions and physical descriptions that were matched for valence and engagement.

## Results

Intergroup bias in the event + physical condition (*M* = 37.0, *SD* = 29.7) was similar to intergroup bias in the event + mental condition (*M* = 33.0, *SD* = 32.6): a 2 group (in-group, out-group) x 2 description type (event + physical, event + mental) mixed ANOVA, with group as a within-subjects factor, revealed the expected main effect of group on identification scores, (*F*(1,185) = 236.1, *p* < 0.001, η^2^ = 0.56, but there were no other significant main effects or interactions (*F*s < 2.5, *p*s > 0.10).

**Fig 4:**
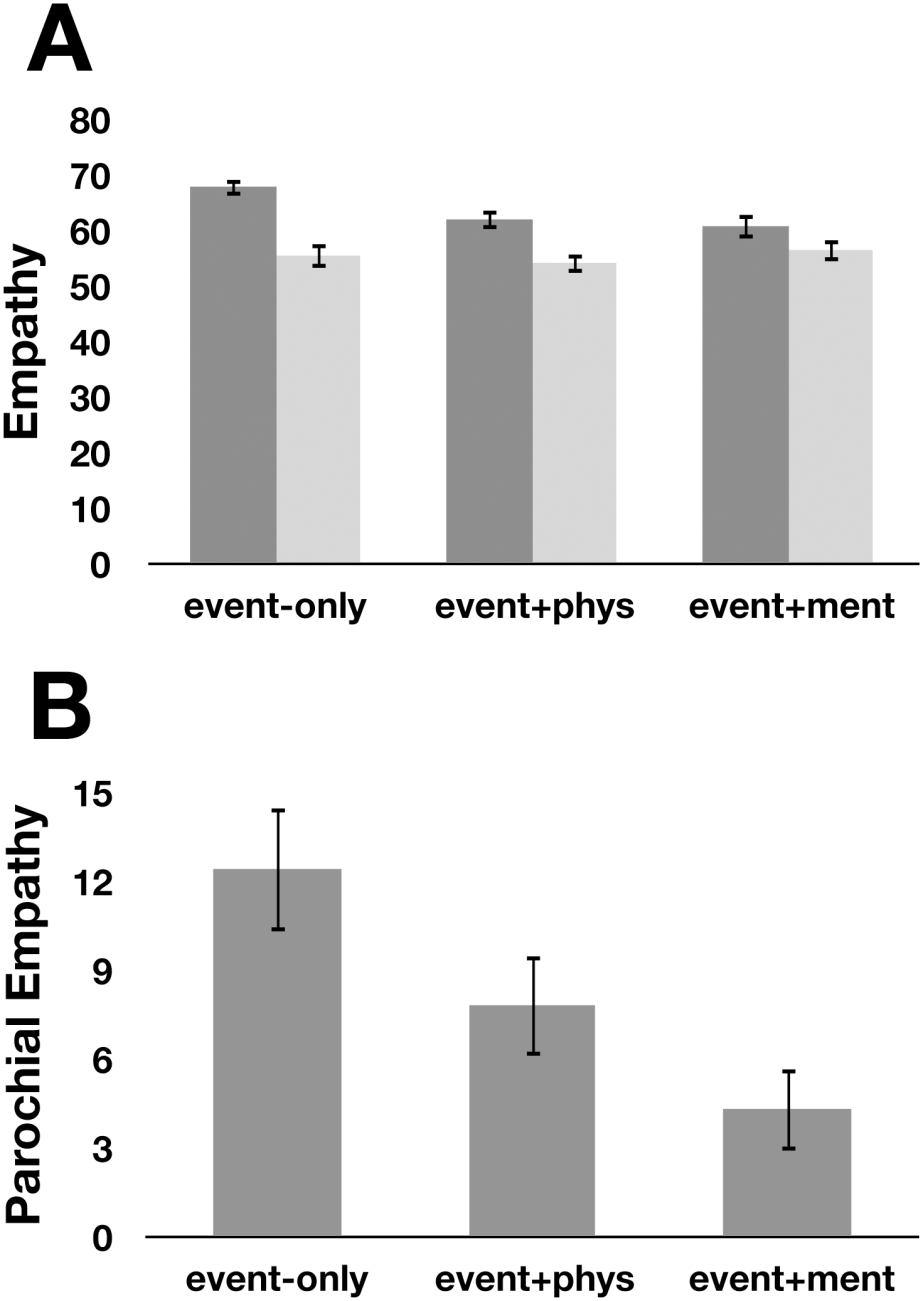
Descriptions of mental states have the greatest impact on parochial empathy (Experiment 3). **(A)** Empathy reported towards in-group and out-group targets when participants were provided only with the events that befell each target (event-only condition, data from Experiment 1), when they were provided with descriptions of physical characteristics of the target prior to the event (event + physical), or when they were provided with emotional and mental state content of the target (personality characteristics, hopes, aspirations) prior to the event (event + mental). **(B)** Parochial empathy from the data in (A). Note that these data include the full set of stimuli; see text for results by stimuli matched for valence and engagement.

The main hypothesis in Experiment 3 was that descriptions of mental states would be most effective at mitigating parochial empathy. To test this prediction, we compared parochial empathy across the event + physical and event + mental conditions. Empathy judgments were analyzed separately for the subset of stimuli matched by valence and the subset of stimuli matched by engagement using 2 group (in-group, out-group) x 2 stimulus type (event + physical, event + mental) mixed ANOVAs, with group as a within-subjects factor (see Table 2 for mean responses by condition). For valence-matched stimuli, there was a significant main effect of group (*F*(1,185) = 21.7, *p* < 0.001, η^2^ = 0.11) (consistent with Experiments 1 and 2), that was qualified by the predicted group x stimulus type interaction (*F*(1,185) = 5.3, *p* = 0.03, η^2^ = 0.03). There was no main effect of stimulus type (*F*(1,185) = 0.0, *p* = 0.95). For engagement-matched stimuli, there was also a significant main effect of group (*F*(1,185) = 19.3, *p* < 0.001, η^2^ = 0.09), and the predicted group x stimulus type interaction was marginally significant (*F*(1,185) = 3.7, *p* = 0.055, η^2^ = 0.02). There was no main effect of stimulus type (*F*(1,185) = 0.2, *p* = 0.65). Overall, parochial empathy in the event + mental condition (engagement-matched: *M* = 3.4, *SD* = 17.3; valence-matched: *M* = 3.1, *SD* = 16.9) was less than half the parochial empathy reported in the event + physical condition (engagement-matched: *M* = 8.8, *SD* = 20.7; valence-matched: *M* = 8.7, *SD* = 17.9). Simple effects analyses revealed that empathy reported while reading the stimuli in the event + mental condition was not significantly greater for targets from the in-group (*M* = 62.2, *SD* = 22.3) versus the out-group (*M* = 58.8, *SD* = 22.8; *t*(92) = 1.9, *p* = 0.059) for the stimuli matched by engagement, and empathy was also not greater for targets from the in-group (*M* = 61.2, *SD* = 21.3) versus the out-group (*M* = 58.1, *SD* = 22.1; *t*(92) = 1.8, *p* = 0.079) for the stimuli matched by valence. On the other hand, empathy reported in the event + physical condition was significantly greater while reading about targets from the in-group (*M* = 63.6, *SD* = 17.5) versus the out-group (*M* = 54.8, *SD* = 18.1; *t*(93) = 4.1, *p* < 0.001, *d* = 0.49) for the stimuli matched by engagement, and empathy was also greater for targets from the in-group (*M* = 63.9, *SD* = 17.4) versus the out-group (*M* = 55.1, *SD* = 18.5; *t*(93) = 4.7, *p* < 0.001, *d* = 0.49) for the stimuli matched by valence (Fig 4).

## Discussion

Consistent with our predictions, these data indicate that describing others’ minds (versus others’ physical characteristics) is more effective for reducing parochial empathy. Since the mental and physical descriptions each contained the same number of words, the effect of narrative description type on parochial empathy is likely due to the specific content of the narratives, rather than a dilution of parochialism by the amount of extraneous information. The effect of descriptive content on parochial empathy was also not due to the valence or engagement of the stories, as the subset of physical descriptions and mental descriptions that did not differ across these dimensions still elicited markedly different parochial empathy.

## General Discussion

Across three experiments, and consistent with previous work using the same paradigm [6], we find that in a competitive context, participants report more empathy for novel in-group than out-group targets. Converging with extensive research on minimal and arbitrary groups [33, 34], these results illustrate that a history of violence and aggression are not necessary to induce empathic differentiation across group boundaries. However, relatively simple interventions involving short individuating information can mitigate parochial empathy. Narrative descriptions decrease parochial empathy not through enhancing overall empathy, but through disrupting parochialism—specifically, by shifting focus away from a target’s group membership and towards individuating information [8, 35], and particularly towards that target’s mind.

Parochial empathy appears to be robust and consistent, and initiated in groups with no history of violence or conflict. Nevertheless (and encouragingly) it is also flexible, and can be decreased by reading short descriptive narratives about each individual. The power of narratives to change attitudes has been illustrated previously, as short narratives about individuals have been used to explicitly increase empathy towards the group that they represent (e.g. women with AIDS, the homeless, African Americans, convicted felons) [17, 18, 36]. For example, White college students who read or listened to short narratives about a woman with AIDS or a convicted murderer with explicit instructions to empathize with the character subsequently reported more empathic concern (compassion, warmth, kind-heartedness) towards the group as a whole, compared to participants instructed to read the story “objectively” [17].

The current results extend these prior findings in at least two ways. First, previous studies using narratives as experimental manipulations focused on stigmatized and remote out-groups that pose little direct threat to the participants [17, 18]. Finding that narratives can reduce parochial empathy specifically in the context of inter-group, zero-sum competition potentially extends the relevance of narrative-based interventions to groups in more direct competition. Second, most prior research focuses on a comparison between two sets of explicit instructions: instructions to “empathize” versus to “respond objectively”. Differences between these conditions leave open the question of whether instructions to empathize increase empathy, or instructions to react “objectively” decrease empathy (e.g. by causing participants to objectify the target of the stories). A particular strength of the current paradigm is the induction of empathy spontaneously, without any overt instructions to empathize or take the character’s perspective. In inter-group conflict, it may be especially important to avoid defensive reactions. Overt instructions to empathize may be aversive and create a defensive backlash, for example by activating meta-stereotypes (i.e. fears about the out-group’s attitude towards the in-group [37]). By contrast, narrative descriptions may act as a relatively unthreatening invitation to passive perspective-taking, which therefore may disrupt defensiveness against unfamiliar or counter-attitudinal ideas [38].

To examine the mechanism by which narrative descriptions decrease parochial empathy, we used a surprise memory test. This task follows a tradition in social psychology of using subsequent memory errors to examine group representations. For example, in the ‘category confusion task’ [25], people first watch group discussions among heterogeneous groups, and then are asked to recall which individual made which statement; memory errors (i.e. mis-assignment of statements) are more common across individuals who share a group category (e.g. gender or race). Higher intragroup mis-attribution of statements is taken to be a measure of perceived group ‘entitativity’ [35]. Although the specific dimensions of analysis in the present study (group membership or events) were somewhat distinct from the ‘category confusion task’, the principle of the memory task was similar: to infer aspects of the initial group representation from subsequent memory errors. We found that errors about group membership were greater in the descriptive information condition, suggesting that group information was less attended to when reading descriptions. Also, memory for group mediated the effect of the descriptions on parochial empathy. Importantly, the increased group memory errors following individual descriptions were not due solely to increased memory load, because the same stimuli led to *better* memory for the sentence describing the specific event.

While descriptive information was able to cause a shift in attention from groups to individuals, this effect depended upon the content of the narratives. Rather than uniformly diluting parochial empathy, the content of the descriptions modulated their effectiveness. The research extends this ‘dilution effect’ literature in a number of ways: first, we focus specifically on empathy, rather than out-group stereotypes. Second, we illustrate a new mechanism by which the shift from group- to individual-level focus occurs—presenting short narrative descriptions about others. One interesting possibility is that narrative descriptions are particularly effective at decategorizing others because (according to our memory data) they both push targets *away* from group-level characteristics, and also draw them *towards* individual-level characteristics. Finally, ‘dilution effects’ on stereotypes are generally shown to be more effective if they are socially relevant [39]. However, a systematic investigation into the types of information that may maximize the dilution effect is missing. Here, we tapped the findings from cognitive neuroscience [31, 40, 41] and social psychology [13, 30, 32] suggesting that humanization is intrinsically linked to mind attribution. We tested directly the hypothesis that descriptions of others’ minds would be particularly effective at eroding parochial empathy, and show that descriptions highlighting targets’ personalities, hopes, thoughts and dreams are more effective at decreasing parochial empathy than equally positive and engaging descriptions of targets that lack these mental state descriptions.

With these data come a series of open questions about the possible scope and application of narrative descriptions as an intervention, such as: (1) whether longer descriptions could eliminate (rather than just decrease) parochial empathy, (2) if the effect of narratives on parochial empathy can endure over time, (3) if descriptions can mitigate parochial empathy in groups in real conflict, or with a history of hostility, or negative stereotypes, (4) how the cultural (in)appropriateness of the information might increase or decrease parochial empathy, and (5) how important the structure of the information (i.e. a narrative format versus a bullet point description). It would also be interesting to examine how parochial empathy acts when a narrative refers to a group rather than to individual group members. These questions should be addressed in future research.

### Summary

Across experiments, a broad characterization of parochial empathy emerged from the studies presented here: we found that parochial empathy can be decreased through the targeted use of short descriptions (Experiment 1), by drawing focus away from group membership (Experiment 2), and towards the minds of the target individuals (Experiment 3).

We do not suggest that interventions aimed at improving global or trait empathy are fruitless, or that narrative descriptions do not (or cannot) increase overall empathy. It seems likely that immersive narratives have the potential to open people up to alternate explanation of past events, or help them to empathize with the experiences of in-group and out-group members. Instead, the present study suggests that narrative descriptions may be best used to mitigate parochial empathy not by increasing empathy, but by decreasing parochialism.

## Supporting Information

S1 File Stimuli for all conditions, across all studies. (DOCX)

S2 File The impact of narratives on unaffiliated targets. We examined responses to the same stimuli used in Experiment 1, but associated with individuals outside of any group affiliation. Responses were either provided for how bad/good participants felt in response to the fortunes/misfortunes, or how good/bad participants thought the protagonists in the scenarios felt.
(DOCX)

S1 Table Narrative descriptions decrease parochial empathy in ID-post participants. Parochial empathy was examined in participants who rated in-group and out-group identification ratings after making in-group and out-group empathy judgments (ID-post) using a 2 target group (in-group, out-group) x 2 severity (mild, extreme) x 2 narrative (event-only, event+narrative) ANOVA, with target group as a within-subjects factor.
(DOCX)
