# Supplementary material for: Minding the Gap: Narrative Descriptions about Mental States Attenuate Parochial Empathy
Source: PLoS One. 2015 Oct 27;10(10):e0140838. doi: 10.1371/journal.pone.0140838 (PMC4624695; doi:10.1371/journal.pone.0140838)
Supplement: S1 File — (DOCX) [file pone.0140838.s001.docx]

**S1 File. Stimuli for all Experiments.**

**Mild, Event-Only**

*Misfortunes*

1. Andrew sat in gum on a park bench.
2. Laura lost $5.
3. Brendan accidentally walked into a glass door.
4. Diana had a stomach ache after her lunch.
5. George stubbed his toe.
6. Lydia missed the bus, which left right as she arrived at the station.
7. Bryan got soaked by a taxi driving through a puddle.
8. Melanie stepped in dog poo.

*Fortunes*

1. Bill found a $5 bill on the street.
2. Jane managed to get indoors before it started pouring rain.
3. Ted found a sentimental possession he thought he had lost.
4. Liane ate a really good sandwich.
5. Jack won a bet.
6. Kristen found money in the coin return slot of a phone.
7. Kurt happened upon a free concert in the park.
8. Kerri caught the bus because it got stuck at a red light.

**Extreme, Event-Only**

*Misfortunes*

1. Andrew sat on a stray nail on a bench.
2. Laura lost her wedding ring.
3. Brendan walked into a glass door and broke his nose.
4. Diana got the H1N1 flu and was very ill.
5. George broke his foot.
6. Lydia missed her flight to her best friend's wedding.
7. Bryan lost a week of work when his computer crashed.
8. Melanie had a stranger throw up on her.

*Fortunes*

1. Bill found $500 on the street.
2. Jane just missed being hit by a bus.
3. Ted recovered all his data when his computer crashed.
4. Liane became a new aunt.
5. Jack won a national championship.
6. Kristen was given a large donation for her non-profit.
7. Kurt got a free backstage pass to his favorite band.
8. Kerri was late for a flight, but made it because it was delayed.

**Mild, Narratives+Events**

*Misfortunes*

1. Andrew recently graduated and has been working hard at his first job. Andrew is in the same position that he started in, and is hoping to be promoted soon. Most days Andrew goes with his friend to the park for lunch. One day Andrew sat on a park bench while talking with his friend and sat directly on some gum. Andrew stood up and looked down to see gum on his pants.
2. Laura is excited to have finally gotten engaged. Laura's engagement ring is a family heirloom and is so beautiful to her. Stepping out of a cab to meet with a wedding caterer, Laura's purse caught on the door and some money slipped out of her wallet. Laura watched as a $5 bill fell into a storm drain. She looked for it briefly, but the heavy rains had washed it into the sewers and it was lost.
3. Brenden has been renting places for many years, but his work is going well and he is finally at a place where he can buy his own home. During his lunch hour, Brenden squeezed in a look at a new house on the market. As he was rushing to check out the back yard, Brenden didn't notice the sliding glass door. He walked straight into the glass door, leaving a smudge on the glass.
4. Diana has always been a dreamer. When she was a child, she wanted to fly in space and be president; now Diana dreams of getting her pilot's license and becoming fluent in at least 3 languages. Diana travels often, and went to China recently. While there, she ate at a recommended restaurant. Diana was very hungry and wolfed down her food. After lunch, Diana had a stomachache.
5. George has always wanted to have a positive effect on the world. After graduating from college, he began teaching in an inner-city elementary school. George played basketball in high school, and since he is eager to connect with his kids he began playing with some of the older kids on the playground and helping them with their game. After jumping up for a rebound, George came down awkwardly and hurt his toe.
6. Lydia and her best friend have talked about being at each other's weddings from when they were children. Now Lydia's friend is getting married in a small town on the other side of the country and Lydia is the maid-of-honor. The week before the wedding Lydia went on her lunch hour to try on her bridesmaid's dress. She went outside, but just missed her bus, which left right as she arrived.
7. Bryan is recently married and he is excitedly expecting the birth of his first child soon. Bryan has been working hard at a brand new job and is hoping to impress his boss so he can feel comfortable taking time off for paternity leave. Bryan worked furiously, trying to finish a big project before his child arrived. After his last day of work as he was walking to his car, Bryan got soaked by a taxi driving through a puddle.
8. Melanie was looking forward to going to college. Melanie lived in the city and was taking a bus to meet with an interviewer for one of the schools she applied to. She was anxious on the bus and didn't notice where she was going when she stepped off. Just off the bus, after she had slung her bag over her shoulder, Melanie stepped in some dog poo.

*Fortunes*

1. Bill was a great athlete in high school, but has had little time to work out since his children were born. Now Bill aspires to become more physically fit: he thinks it's important since he wants to be able to run around endlessly with his kids. On his way to work, Bill saw something under some cardboard in the alley. He reached down and found a $5 bill.
2. Jane was hoping to make more money by taking on another job, since her finances were tight. She is very good with crafts and was lost in thought walking home from work about how she might be able to sell these online. As Jane was crossing the street, she looked up to see that the clouds were threatening rain. Jane managed to get indoors before it started pouring rain.
3. Ted is at the age where he is ready to settle down: he wants to meet his soul-mate and get married. Ted has now been prioritizing dating people, but his demands at work are great. Just as Ted was heading out from work to meet a date he was very excited about, he checked for a sentimental possession that he had lost weeks ago. Just then, he found it on the floor underneath a filing cabinet.
4. Liane has dreamed of owning her own home so that her family can easily visit, and after many years of working hard she finally has it. Liane was waiting in her new home to hear from her family, who were coming to visit. Liane finally got a call: they were on the road and would be there by dinner. Liane sat down and ate a really good sandwich for lunch.
5. Jack grew up as the youngest brother in a large family. He loves his family and wants to have 4 or 5 of his own children one day. One of the things that Jack likes best about his family is the support that he always gets from them, and the small competitions that he has with his older brothers. Recently, something happened to him that had never happened before: against his oldest brother, Jack won a bet.
6. Kristen has been busy since graduating from college: she opened a non-profit company that trains people from poor communities around the world in sustainable farming techniques. Kristen also plans to become a doctor one day, but not until her company is stable enough to run without her constant attention. Outside of the office is an old payphone. Recently Kristen found some money in the coin return slot of the phone.
7. Kurt has cousins who live on a ranch, and he has always wanted to own his own horse. Kurt has been on many rides with his cousins, and those times have been some of the best of his life. His cousins also like visiting him in the big city, and are there at his place often. On a walk with his cousins during one of their recent visits, Kurt happened upon a free concert in the park.
8. Kerri has enjoyed raising her children, but she misses having babies around. It surprised her how much she loved babies when she had them herself. Now Kerri is looking forward to having many grandchildren that she can play with and spoil. Her oldest daughter just had a child and Kerri was taking the bus to see them. When Kerri arrived at the bus stop, she thought she had missed it, but she ended up catching the bus because it got stuck at a red light.

**Extreme, Narratives+Events**

*Misfortunes*

1. Andrew recently graduated and has been working hard at his first job. Andrew is in the same position that he started in, and is hoping to be promoted soon. Most days Andrew goes with his friend to the park for lunch. One day Andrew sat on a park bench while talking with his friend and sat directly on a stray nail. Andrew jumped up and looked down to see blood on the rusty nail.
2. Laura is excited to have finally gotten engaged. Laura's engagement ring is a family heirloom and is so beautiful to her. Stepping out of a cab to meet with a wedding caterer, Laura's ring caught on the door and slipped off her finger. Laura watched as the ring bounced into a storm drain. She had the city open the drain, but the heavy rains had washed it into the sewers and it was lost.
3. Brenden has been renting places for many years, but his work is going well and he is finally at a place where he can buy his own home. During his lunch hour, Brenden squeezed in a look at a new house on the market. As he was rushing to check out the back yard, Brenden walked straight into the sliding glass door, breaking his nose. Brenden went back to work with blood all over his shirt.
4. Diana has always been a dreamer. When she was a child, she wanted to fly in space and be president; now Diana dreams of getting her pilot's license and becoming fluent in at least 3 languages. Diana travels often, and went to China recently. While there, she got the H1N1 flu. Diana found herself terribly ill, quarantined in a foreign country, and hearing reports of people dying from the illness.
5. George has always wanted to have a positive effect on the world. After graduating from college, he began teaching in an inner-city elementary school. George played basketball in high school, and since he is eager to connect with his kids he began playing with some of the older kids on the playground and helping them with their game. After jumping up for a rebound, George came down awkwardly and broke his ankle.
6. Lydia and her best friend have talked about being at each other's weddings from when they were children. Now Lydia's friend is getting married in a small town on the other side of the country and Lydia is the maid-of-honor. On the morning of the wedding Lydia got delayed at work and was late to the airport, missing the flight. She was told that the next flight didn't arrive until the wedding was over.
7. Bryan is recently married and he is excitedly expecting the birth of his first child soon. Bryan has been working hard at a brand new job and is hoping to impress his boss so he can feel comfortable taking time off for paternity leave. Bryan worked furiously, trying to finish a big project before his child arrived. Just before his child's due date, Bryan's computer crashed. A full week of work was completely lost.
8. Melanie was looking forward to going to college. Melanie lived in the city and was taking a bus to meet with an interviewer for one of the schools she applied to. She was anxious on the bus and didn't notice when the person next to her began holding his stomach. Suddenly, the man next to her threw up all over her. She was far from home and her interview started in an hour.

*Fortunes*

1. Bill was a great athlete in high school, but has had little time to work out since his children were born. Now Bill aspires to become more physically fit: he thinks it's important since he wants to be able to run around endlessly with his kids. On his way to work, Bill saw something under some cardboard in the alley. He reached down and pulled up a bunch of soggy $20 bills: almost $500 total.
2. Jane was hoping to make more money by taking on another job, since her finances were tight. She is very good with crafts and was lost in thought walking home from work about how she might be able to sell these online. As Jane was crossing the street, she looked up to see that she was crossing against a red light. Jane stepped back just in time, and a bus narrowly missed hitting her.
3. Ted is at the age where he is ready to settle down: he wants to meet his soul-mate and get married. Ted has now been prioritizing dating people, but his demands at work are great. Just as Ted was heading out from work to meet a date he was very excited about, his computer crashed. He went to the IT department, and they were able to save all of the data from his hard drive.
4. Liane has dreamed of owning her own home so that her family can easily visit, and after many years of working hard she finally has it. Liane was waiting in her new home to hear from her family: her sister had just gone into labor. Liane finally got a call: it had all gone well and she was now a brand new aunt. Liane cried with happiness when she got off the phone.
5. Jack grew up as the youngest brother in a large family. He loves his family and wants to have 4 or 5 of his own children one day. One of the things that Jack likes best about his family is the support that he always gets from them. Recently, Jack was competing at the collegiate level in wrestling. His whole family was at the final meet to see him, and Jack won the national championship.
6. Kristen has been busy since graduating from college: she opened a non-profit company that trains people from poor communities around the world in sustainable farming techniques. Kristen also plans to become a doctor one day, but not until her company is stable enough to run without her constant attention. Last week Kristen came one step closer to her dream when her non-profit company received its largest ever donation.
7. Kurt has cousins who live on a ranch, and he has always wanted to own his own horse. Kurt has been on many rides with his cousins, and those times have been some of the best of his life. His cousins also like visiting him in the big city, and are there at his place often. Recently Kurt was able to give them a special treat: he won tickets to their favorite band.
8. Kerri has enjoyed raising her children, but she misses having babies around. It surprised her how much she loved babies when she had them herself. Now Kerri is looking forward to having many grandchildren that she can play with and spoil. Her first child just went into labor and Kerri was flying out to be there for the delivery. Terrible traffic made Kerri late; the plane was delayed, though, so Kerri still made the flight.

**Mild, Mental+Events**

*Misfortunes*

1. Andrew is serious and quiet. He is the type of person who focuses his mind on big projects and finishes them, but is often forgetful about little things (like where his phone is). Andrew is looking forward to being a father one day. Recently, Andrew sat on gum on a park bench.
2. Laura is quiet and reserved, and is often pulling things apart to see how they work. She is a very analytical thinker and makes very rational decisions in life. Laura loves children and is looking forward to having another soon. Recently, Laura lost $5.
3. Brendan thinks of other's needs often, sometimes forgetting his own. He likes to people-watch and imagine other people's lives. Andrew has been unlucky in relationships and is hoping to find someone he can fall in love with. Recently, Brendan walked into a sliding glass door.
4. Diana is open-minded and sensitive to other people's beliefs and feelings. Diana is the type of person who appreciates having her assumptions be challenged by art. Diana dreams often about owning a small house of her own. Recently, Diana had a stomachache.
5. George is pretty intuitive about other people. He likes to do things his own way and think up unique solutions to problems. George someday hopes to go back to school to get a degree so he can work in a job he loves. Recently, George stubbed his toe.
6. Lydia has strong beliefs and is very concerned about people and the environment. She is very laid-back, except when her core values are challenged. When she was a girl, she dreamed of traveling, and hopes to go to Africa soon. Recently, Lydia just missed her bus, which left right as she arrived.
7. Bryan makes lots of lists and loves checking things off. He likes to learn and to plan and complete projects, but only if they are done well. He has always been an athlete, and his dream is to win a national championship in diving. Recently, Bryan got soaked by a taxi driving through a puddle.
8. Melanie values creative thinking and loves ideas. She enjoys reading from a diverse set of thinkers, and likes to make connections between different ideas. She has always wanted to learn more about philosophy and is finally taking a class. Recently, Melanie stepped in some dog poo.

*Fortunes*

1. Bill is much more fond of doing than watching. When he learns something, he likes to throw himself in there and try it right away. He enjoyed building models as a kid, and is now hatching a scheme to re-build an old car. Recently, Bill found a $5 bill on the street.
2. Jane is very practically-minded. She often wonders how people can follow ideas that have not been shown to work in the past. Jane has also been training in Karate, and rehearses her routines in her mind over and over again. Recently, Jane managed to get indoors before it started pouring rain.
3. Ted is fun-loving and likes to help others enjoy the moment. He is very street smart and able to read people's intentions well. Ted is excited about an upcoming school reunion and is dreaming up a plan to get all his old friends together. Recently, Ted found a sentimental possession he thought he had lost.
4. Liane has a strong sense of responsibility and duty. She likes to be liked, and often puts others' needs in front of her own. Liane is going back to school to be a social worker, where she hopes to help people through their problems. Recently, Liane ate a really good sandwich.
5. Jack has a broad range of interests and is excited by new experiences. He is more of an ideas person, and is sometimes bored with the details. Jack aspires to be on a game show and signs up to be in the audience regularly. Recently, Jack won a bet.
6. Kristen cares a lot, sometimes maybe too much, about how other people feel. She is good at seeing the world through other people's eyes. Kristen recently started online dating and spends her time trying to decode people's real intentions. Recently, Kristen found money in the coin return slot of a vending machine.
7. Kurt says what he thinks and enjoys debating other people. He likes being surrounded by other people - both old friends and strangers on the street. He is not particularly happy in his current job and is thinking about a job where he can work more with people. Recently, Kurt happened upon a free concert in the park.
8. Kerri likes to understand difficult problems and create solutions. She stays well-informed and enjoys managing other people. Kerri has worked hard at her current job and has been thinking about how to position herself for a promotion. Recently, Kerri caught the bus because it got stuck at a red light.

**Mild, Physical+Events**

*Misfortunes*

1. Andrew looks to be in his 40s. He is tall with short, dark hair and a well-trimmed beard. He is at the beach and his hair is being tussled by the wind. He wears a sweatshirt and light coat, which hang off his bulky shoulders. Recently, Andrew sat on gum on a park bench.
2. Laura looks to be in her 30s. She has olive skin and dark, curly hair, which is held back in a braid high on her head. She has large, brown eyes and high, arching eyebrows. She is wearing golden hoop earrings that reflect the light of a sunset, and is wearing sunglasses. Recently, Laura lost $5.
3. Brendan looks to be in his 30s. Brendan has short, red hair. He has freckles on his face, and red stubble on his chin. Brendan is sitting at a table with hands folded under his chin. He is looking down under his wire-rimmed glasses wearing a faint smile. Recently, Brendan walked into a sliding glass door.
4. Diana looks to be in her mid-20s. She has short dark hair, which is spiked up on her head. Her long, oval face has high cheek bones, and she wears a pair of colorful glasses. On her thin lips is bright lipstick. Recently, Diana had a stomachache.
5. George looks to be in his 50s. He has blonde hair, that peeks out in tufts from a hat that shades all but a faint smile on his face. He is wearing a red shirt that brings out the slight sunburn on his thick neck. Recently, George stubbed his toe.
6. Lydia looks to be in her 40s. She has shoulder-length, blond hair, which falls down in front of her face. Her bright blue eyes are framed by dark lashes and thin eyebrows. She has a scarf draped casually over her shoulders. Recently, Lydia just missed her bus, which left right as she arrived.
7. Bryan looks to be in his 20s. He has thick, dirty-blond hair and a long, slender face with very pale eyes. He is wearing a tank top and his long arms are spread out to the side. Recently, Bryan got soaked by a taxi driving through a puddle.
8. Melanie looks to be in her 20s. She has straight, blond hair that hangs down past her shoulders. She has a round face, a small nose and dimples in her cheeks. She is wearing shorts and a t-shirt with the sleeves rolled up. Recently, Melanie stepped in some dog poo.

*Fortunes*

1. Bill looks to be in his 40s. He has wild, unruly hair, which falls down over his forehead. His skin is leathery and dark, and is wearing a flannel shirt tucked in to brown pants. He has dark eyes set deep beneath bushy eyebrows. Recently, Bill found a $5 bill on the street.
2. Jane looks to be in her 30s. She has shoulder length hair that is parted on the side. Her fine and glossy hair is tucked back behind her ears. She has on a winter coat that reaches to her boots, and her arm is held casually at her side. Recently, Jane managed to get indoors before it started pouring rain.
3. Ted looks to be in his 40s. He has short, brown hair that is tightly curled. His long, sloping forehead comes down to meet bushy eyebrows and smile lines around his dark eyes. He is standing with his shoulders slightly hunched forward. Recently, Ted found a sentimental possession he thought he had lost.
4. Liane looks to be in her 20s. She has shiny black hair, and thin, drawn lips. Her almond eyes slant down slightly at the corners. She is standing in the shade of a tree, with a grass lined path stretching back behind her into the distance. Recently, Liane ate a really good sandwich.
5. Jack looks to be in his early 30s. Jack is stocky and his head is shaved bald. His beard is shaped into a dark goatee. He stands in front of a very old building. Candles burn in the background. He has a square jaw, and full lips that are pulled into a smile. Recently, Jack won a bet.
6. Kristen looks to be in her 20s. She has long, dark hair parted in the middle. Her hair frames her face. She is wearing a large sun hat, and she her mouth is open. Her small ears have many earrings studding them, and wears a necklace. Recently, Kristen found money in the coin return slot of a vending machine.
7. Kurt looks to be in his 20s. He has clean-cut hair and a smoothly shaved face. He is skinny, with strong, hairy arms. A faint scar running along his jaw is visible as he works on something at the table in front of him. Recently, Kurt happened upon a free concert in the park.
8. Kerri looks to be in her 50s. She has a round face and silver and black hair that is bobbed above her shoulders. She is wearing a flowery top with a high neck line. She has large glasses and a small cleft in her chin. Recently, Kerri caught the bus because it got stuck at a red light.
