# Supplementary material for: Minding the Gap: Narrative Descriptions about Mental States Attenuate Parochial Empathy
Source: PLoS One. 2015 Oct 27;10(10):e0140838. doi: 10.1371/journal.pone.0140838 (PMC4624695; doi:10.1371/journal.pone.0140838)
Supplement: S2 File — We examined responses to the same stimuli used in Experiment 1, but associated with individuals outside of any group affiliation. Responses were either provided for how bad/good participants felt in response to the fortunes/misfortunes, or how good/bad participants thought the protagonists in the scenarios felt. (DOCX) [file pone.0140838.s002.docx]

**S2 Experiment 1b. The impact of narratives on unaffiliated targets.**

Experiment 1b was designed primarily to illuminate the unexpected finding, in Experiment 1a, that adding narratives tended to decrease overall levels of empathy. The narratives described a sequence of events from the perspective of the protagonist; these life events often also provided a window on the larger landscape of target’s lives (“John comes from a large family…”, “Bryan is expecting his first child…”). We hypothesized that the narrative descriptions contextualized the specific fortunes/misfortunes described by the events. That is, the descriptions may have made the specific fortunes/misfortunes described by the events seem relatively trivial in the larger scope of life. If so, this hypothesis predicts that after reading the descriptions, observers would expect the *targets themselves* to have more muted (proportionate) emotional reactions to the fortunes/misfortunes. Furthermore, this overall effect of descriptions should occur independent of the group context, i.e. even for targets who are unaffiliated with either group.

**Method**

Participants in Experiment 1b (*n* = 920) were provided with the same scenarios presented in Experiment 1a, but there was no group membership information provided. Participants responded to each scenario either by rating how they thought the target of the story felt, or how they felt for the target. Participants were assigned to one or the other of these conditions, and data were collected in two waves at approximately the same time of day (~ 12:00 pm on a weekday); order of collection for each condition was counterbalanced across waves. Within a condition, participants were randomly assigned to view 1 of the 4 different stimulus sets (event-only versus event + description, mild versus severe). Experiment 1b was therefore a 2 task (rating the target’s own emotion versus rating the participant’s empathy) x 2 stimulus type (event-only versus event + description) x 2 event severity (mild versus extreme) between subjects design. Based on the catch scenario, data from 83 participants were eliminated, resulting in 400 included participants in the ‘target emotion’ task (219 female, *M*_age_ = 34.5, *SD* = 12.1) and 437 included participants in the ‘participant empathy’ task (187 female, *M*_age_ = 31.0, *SD* = 11.2). Participants were not allowed to participate in more than one version of the study; no participants attempted to do so.

*Experimental design*

*Stimuli*. Stimuli were identical to Experiment 1a.

*Procedure.* For Experiment 1b, no personality questions were asked, no groups were introduced, and the events were presented on the screen without the team logos. In both conditions, participants were told the following: “We have collected some information from a number of people, including an event that happened to them recently, and we now want to see how others respond to the information.” Participants in the ‘target emotion’ condition were then told, “You will be reading 17 brief stories/events and using slider bars to indicate how you think each character in the following stories felt,” while participants in the ‘participant empathy’ condition were told, “You will be reading 17 brief stories/events and using slider bars to indicate how reading each of the stories/events made you feel.” The targets were therefore unaffiliated with respect both to any experimental manipulation (i.e. “Eagles” and “Rattlers”), and to the subject recruitment database (i.e. “mTurkers”). As with Experiment 1a, each participant saw 16 stimuli, all from only one of the 4 stimulus sets: mild events-only, mild events + narrative, extreme events-only, extreme events + narrative; each condition also presented participants with a final attention check question. Ratings for the unaffiliated targets in the ‘participant empathy’ task were the same as Experiment 1a (“How good/bad do you feel that [event] [target]?”). In the ‘target emotion’ task, participants instead judged “How good/bad do you think [target] feels that [event]?”, from “not at all bad/good” to “extremely bad/good”.

**Results**

To investigate the effects of narrative descriptions on ratings of the targets perceived emotional responses and the participants’ empathy, we analyzed the results for Experiment 1b using a 2 task (‘target emotion’ versus ‘participant empathy) x 2 stimulus type (event-only versus event+narrative) x 2 event severity (mild versus extreme) between-subjects ANOVA.

First, there was a main effect of the task (‘target emotion’ vs ‘participant empathy’, *F*(1,829) = 193.9, *p* < 0.001, η^2^ = 0.19): unsurprisingly, participants reported that the targets’ first-person emotional response to their own fortunes and misfortunes (*M* = 83.4, *SD* = 0.7) would be more intense than the participants’ third-person empathic responses to the same events (*M* = 69.3, *SD* = 0.7).

Second, confirming our prediction, we replicated the main effect of narrative descriptions observed in Experiment 1a (*F*(1,829) = 8.43, *p* < 0.005, η^2^ = 0.01): descriptions reduced *both* ratings of the targets’ own experience in response to the events, and the participant’s empathy for the targets. The effect of the task did not interact with description condition, and the 3-way interaction was not significant. That is, narrative descriptions had the same effect on ratings of own and targets’ affect.


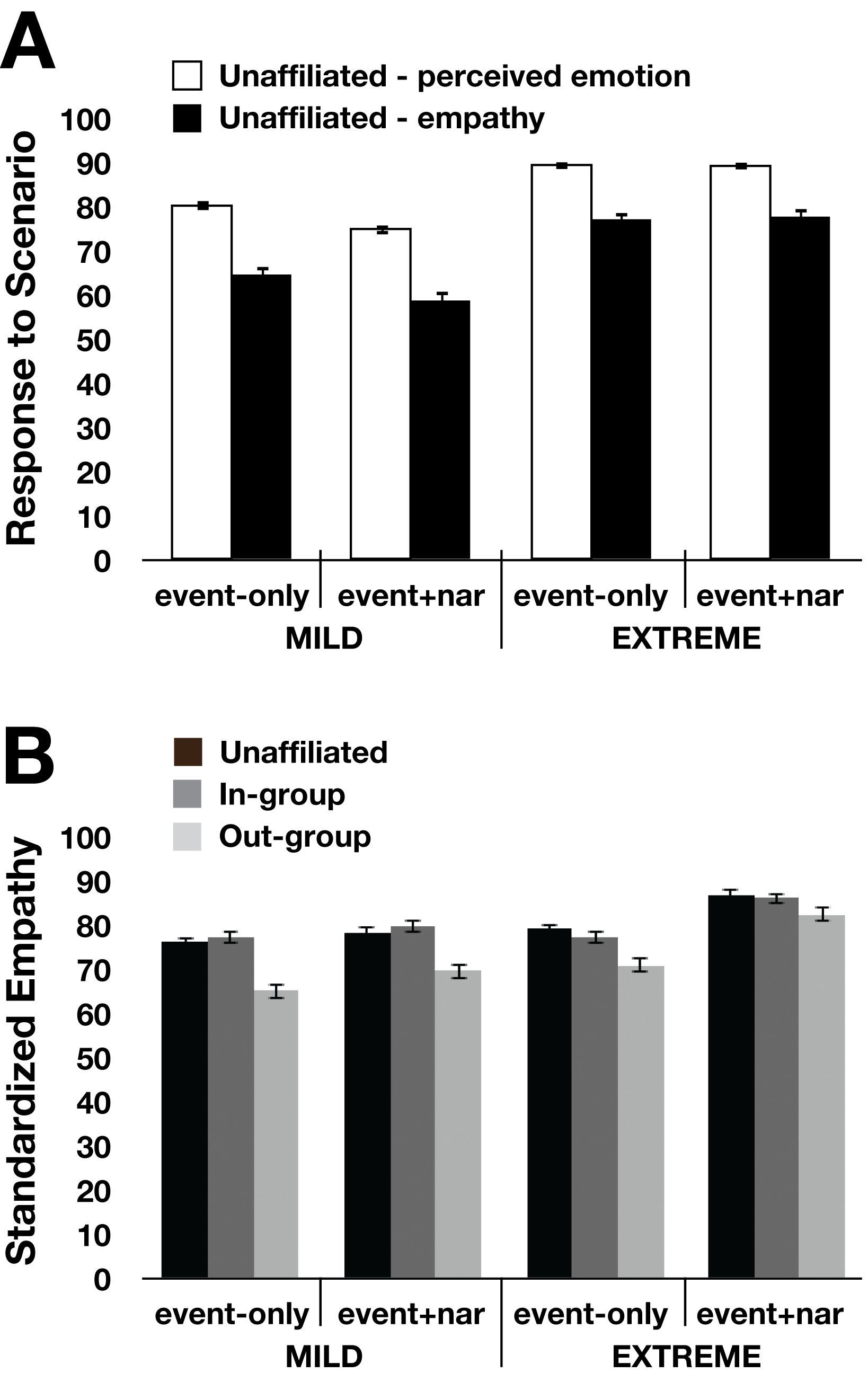
Third, there was a significant stimulus type x severity interaction, *F*(1, 829) = 6.83, *p* < 0.01, η^2^ = 0.01). Follow up analyses revealed a significant main effect of stimulus type on ratings of mild events (*F*(1, 409) = 12.5, *p* < 0.001, η^2^ = 0.03), but no main effect of stimulus type on ratings of extreme events (*F*(1, 420) = 0.05, *p* > 0.80) (Figure 2A). That is, the contextualizing effect of the narrative descriptions, muting emotional responses to the specific fortunes/misfortunes, was particularly true for mild events.

**Figure S1. Perceived first-person experiences and empathic responses to unaffiliated targets (Experiment 1b).** **(A)** Between subjects, participants reported either how bad/good the targets themselves felt (‘unaffiliated emotion’) in response to the fortunes and misfortunes depicted in each of the four conditions (white bars), or how bad/good they felt for the targets (‘unaffiliated empathy’) when the targets were unaffiliated with a group (black bars). **(B)** Narratives close the empathy gap by increasing empathy for out-group members (Experiment 1a) relative to both in-group members (Experiment 1a) and unaffiliated individuals (Experiment 1b), who are indistinguishable from each other by empathy rating across all conditions.

**Discussion**

Replicating the result from Experiment 1a, adding narrative descriptions to events did not result in an overall increase in empathy for those events; in fact, the presence of the descriptions *decreased* both how good/bad the participants thought the targets themselves felt in response to fortunes/misfortunes and how good/bad they felt for the participants. Participants who read narrative descriptions may have weighed the specific events against the broader backdrop of the protagonist’s life, and thus predicted somewhat muted responses to the events when viewed in context (how much empathy you feel for someone losing $5 depends upon the context of the rest of their life – e.g. whether they are down on their luck or born with a silver spoon – which is revealed in the narrative descriptions). This interpretation is consistent with the greater impact of descriptive information on mild events: a positive background may weigh heavier on one’s empathy towards a mild event, whereas an extreme event may loom relatively larger than the description preceding that event (i.e. the pain of missing a best friend’s wedding can only be mitigated so much by other life events).

The effects of descriptions on the target’s perceived emotions were also transferred to similarly muted empathic responses, when participants reported their own empathic responses to the target’s experience. That is, the small overall decrease in responses to narrative descriptions appears to arise from the perception of the target’s own emotion.

Another aspect of the results that is evident in this depiction is that empathy for the in-group is not different from empathy for an unaffiliated target (Figure 2B). Consistent with our previous results [[1](#_ENREF_1)], parochial empathy was driven by out-group derogation (less empathy for out-group relative to in-group or unaffiliated targets), rather than in-group favoritism. Previous reports show that people experience in-group favoritism in minimal group paradigms, especially with regard to resource allocation [[6](#_ENREF_6), [19](#_ENREF_19), [20](#_ENREF_20)], but that group competition and salience can elicit out-group derogation in addition to (or instead of) in-group favoritism [[21-23](#_ENREF_21)]. The current results are consistent with this distinction: in our paradigm, groups were competing for a limited resource (a bonus for winning the “challenge”), and group identity was enhanced by grouping participants (ostensibly) based on their personalities. Under those circumstances, parochial empathy appears to predominantly reflect out-group derogation.
