# Supplementary material for: Minding the Gap: Narrative Descriptions about Mental States Attenuate Parochial Empathy
Source: PLoS One. 2015 Oct 27;10(10):e0140838. doi: 10.1371/journal.pone.0140838 (PMC4624695; doi:10.1371/journal.pone.0140838)
Supplement: S1 Table — Parochial empathy was examined in participants who rated in-group and out-group identification ratings after making in-group and out-group empathy judgments (ID-post) using a 2 target group (in-group, out-group) x 2 severity (mild, extreme) x 2 narrative (event-only, event+narrative) ANOVA, with target group as a within-subjects factor. (DOCX) [file pone.0140838.s003.docx]

**S1 Table. Narrative descriptions decrease parochial empathy in ID-post participants.**

| Effect | Hyp DF | Err DF | F Value | P Value | η^2^ |
| --- | --- | --- | --- | --- | --- |
|  |  |  |  |  |  |
| Target Group (TG) | 1 | 295 | 27.6 | <0.001 | 0.09 |
| Severity (SEV) | 1 | 295 | 30.2 | <0.001 | 0.09 |
| Narrative (NAR) | 1 | 295 | 2.8 | 0.095 |  |
| TG X SEV | 1 | 295 | 0.8 | 0.361 |  |
| TG X NAR | 1 | 295 | 6.6 | 0.010 | 0.02 |
| SEV x NAR | 1 | 295 | 3.7 | 0.056 |  |
| TG X SEV x NAR | 1 | 295 | 0.6 | 0.441 |  |

Parochial empathy in ID-post participants mirrored the effects of parochial empathy in ID-pre participants for all 4 hypotheses in Experiment 1. For ID-post participants, there was a main effect for group that was characterized by parochialism: higher empathy for in-group (*M*=68.0, *SD*=18.6) than out-group (*M*=63.4, *SD*=18.2) (Hypothesis 1). There was a significant group x narrative interaction, such that empathy in the event-only conditions was higher for in-group (*M*=70.6, *SD*=18.9) than out-group (*M*=63.7, *SD*=18.9), but in the event+narrative condition empathy was similar for in-group (*M*=65.3, *SD*=17.9) and out-group (*M*=63.0, *SD*=17.4) (Hypothesis 2). There was a main effect of extremity (Hypothesis 3), which did not interact with target group (Hypothesis 4).
